# Economic impact of HIV/AIDS: a systematic review in five European countries

**DOI:** 10.1186/s13561-014-0015-5

**Published:** 2014-09-16

**Authors:** Marta Trapero-Bertran, Juan Oliva-Moreno

**Affiliations:** University Pompeu Fabra, Department of Economics and Business, Centre for Research on Economics and Health, Ramon Trias Fargas 25-27, Barcelona, 08005 Spain; University Castilla La-Mancha, Facultad de Terapia Ocupacional, Logopedia y Enfermería, Avda. Real Fábrica de Seda, s/n, Talavera de la Reina, Toledo, 45600 Spain; Facultad de Ciencias Jurídicas y Sociales, Análisis Económico y Finanzas, Cobertizo de San Pedro Mártir s/n, Toledo, 45071 Spain

**Keywords:** Economic impact, Costs, VIH/AIDS

## Abstract

The HIV/AIDS disease represent a priority for all health authorities in all countries and it also represents serious added socioeconomic problems for societies over the world. The aim of this paper is to analize the economic impact associated to the HIV/AIDS in an European context. We conducted a systematic literature review for five different countries (France, Germany, Italy, Spain and United Kingdom) and searched five databases. Three types of analyses were undertaken: descriptive statistics; quantitative analysis to calculate mean costs; and comparison across countries. 26 papers were included in this study containing seventy-six cost estimates. Most of the studies analyzed the health care cost of treatment of HIV/AIDS. Only 50% of the cost estimates provided mean lymphocyte count describing the patients’ disease stage. Approximately thirty percent of cost estimates did not indicate the developmental stage of the illness in the patients included. There is a high degree of variability in the estimated annual cost per patient of the treatments across countries. There is also a great disparity in total healh care costs for patients with lymphocyte counts between 200CD4+/mm3 and 500 CD4/mm3, although the reason of variation is unclear. In spite of the potential economic impact in terms of productivity losses and cost of formal and informal care, few studies have set out to estimate the non-medical costs of HIV/AIDS in the countries selected. Another important result is that, despite the low HIV/AIDS prevalence, its economic burden is very relevant in terms of the total health care costs in this five countries. This study also shows that there are relatively few studies of HIV costs in European countries compared to other diseases. Finally, we conclude that the methodology used in many of the studies carried out leaves ample room for improvement and that there is a need for these studies to reflect the economic impact of HIV/AIDS beyond health care including other components of social burden.

## Background

Since the human immunideficiency virus discovery at the beginning of the 80s and its manifestation in the human immunideficiency, it was evident that this could become one of the bigger problems of the XX century [1]. The HIV/AIDS disease not only had and is having a high impact on populations’ health but it represents serious added socioeconomic problems for individuals, families, communities and governments of many countries [2–6].

Although high activity antiretroviral treatment (HAART) supposed a radical treatment in the therapy for the HIV/AIDS, they are not exempt of problems. Since a while ago, there exists cientific evidence of the advers events (gastrointestinal problems, metabolic alterations, nefro-hepatic problems, etc.) present in a high percentage of patients [7–14]. Another important question is that,though the AIDS phase is not developed, the virus atac at the defense level provoques that a high percentage HIV + people are co-infected by the hepatitis C [15]. On another hand, not always the treatments are succesful in controlling the viral burden and the best answer from the CD4 cells, though this is one of the aspects that clearly shows the improvements in the last year therapies [16–19].

In the OMS Europe region, which comprises all countries that belong to Europe, counted down 2,800 deaths during 2008 due to the HIV/AIDS. Since the beginning of the epidemia till 2008, 342,768 people have been diagnosed with AIDS in that region. This implies that around 150,000 people with this diagnostic lived in that area at that time, from 49 countries out of 57 which have reported information about the acumulated HIV/AIDS cases. The number of incident cases was 5,218 people only from those countries belonging to the EU (excluding Austria, Denmark and liechtenstein, for which there were not data available) being. Estonia, Letonia, Portugal and Spain the fourth country with the highest incidence rate. In Europa, the main cause of transmission of new cases was the heterosexual contact whereas in the EU, the main cannal of transmission was the sex among men, with 40% of new cases, followed by the heterosexual contact (29% of cases) and drugs users (6%), being classified 24% of cases by unknown originated cause [20].

HIV is a priority for all health authorities in all countries over the world as one of the main dangers for individuals health. Although the situation for developed and developing countries is quite different, this disease generates quite a lot of pressure in the Health System according to the amount of resources needed. This implies quite a lot of relevant questions for decision makers.

Although economic evaluations of HAART have revealed being a cost-effective treatment compared to the treatments before [21,22], this does not really imply that the cost of these treatments does not worry the decision makers. Indeed, the appearance of new and more expensive drugs highlights the importance for decision makers to study and put more effort into the resources assignation due to the spread of costs generated. The HAART treatment is not considered the solution of this disease, but has increased considerably the expectancy and quality of life from seropositive persons.

From the economic point of view, the introduction of the HAART has supposed an increase in the total treatment cost. Not really because the annual cost of treating is more expensive but because the increase on the life expectancy in patients [12,22–25]. The cost of treating a patient along their life period notably increases [5,26]. However, unlike what happens with other diseases such as ischemic heart disease, stroke, mental illness or Alzheimer's disease, there are few studies of HIV costs in European countries. So, important doubts still existing regarding the annual treatment costs per patient and the heterogeneity of these costs among the different European countries and the impact of those on the health budget. In addition, it is also an element of interest the distribution of the total healthcare costs and the patient cost according the disease stage and the defences (lymphocytes) level across patients.

However, it is also important to highlight that new treatments have produced a relevant change on the job market incorporation of these disease carriers. In a recent paper [6] comparing the studies carried out in high income countries in the 90s decade with those where the HAART use was extended, there were extensive differences regarding the impact in terms of job market. More precisely, studies carried out during the 80s and early 90s in high-income countries pointed out that an HIV diagnosis had a strong impact on labour force participation [27–30]. The therapeutic advances made since the mid 90s have substantially changed this panorama improving the labour participation of HIV + persons [6,31–35].

Despite the improvement in the participation on the job market on recent years, still important the economic impact and social cost acrued in these population group. The accumulated HIV/AIDS cases, the negative consecuences for health, and the high treatment cost remark this area as a primary objective for all governments across countries.

A global review on the cost of HIV stressed the need to increase the evidence published to more accurately estimate the impact of the overall cost of HIV infection/AIDS [3]. The results from this review demonstrates the great effort and interest embodied in the overall estimate of costs of this disease in this last decade in five European countries.

The aim of this paper is to analyze the health care (hospitalization; antiretrovirals and other drugs; laboratory tests; specialists and GP consultations; and others) and non health care (productivity losses and formal and informal care) resources associated to the HIV treatment in an European context. The added value of this new review on HIV costs is the quantitative analysis using cost estimates for five different countries in Europe, allowing for comparisons among them. This imply to compare treatment and social costs associated to HIV across European countries.

## Methods

The systematic literature review was conducted for five different countries: Spain, Germany, France, Italy and United Kingdom. Five databases (Health Economic Evaluations Database (HEED), NHS Economic Evaluation Database (NHS EED), Health Tecnology Assessment (HTA), MEDLINE Ovid and PubMed, and ECONLIT) were searched from their inception to September 2010 using key words: (cost* or economic* or valu* or pharmac* or impact or burden or drug or expenditure* or willingness or employment or resource* or use* or utilization) crossed with (HIV* or AIDS or antirretroviral therapy) in HEED; and, (HIV* or AIDS or antirretroviral therapy) for the NHS EED and HTA searches. In addition, a search in MEDLINE Ovid and Pubmed, and ECONLIT was undertaken with no relevant results, therefore all results from these three databases were desestimated. The search terms used were in English. The search was limited to papers related to humans and no time restriction was applied, thought all papers published after the introduction of the HAART were excluded. Papers in English and Spanish of cost-of illness giving information of the healthcare and non-healthcare costs were included. Papers were excluded if they: were economic evaluations of HIV/AIDS due to the differences of methodology across studies; were review articles; did not calculate the costs for any of the countries of interest for this study; did not contribute information of interest; were published before 1996, when HAART were introduced, dut to the impact on treatment costs they had. Review articles were disregard to avoid duplication of the same estimates in the quantitative analysis. We assume that all cost estimates included in the different review papers were identified by the the systematic literature review conducted in this paper. If there are more than one cost estimate by paper, all of them will be included in the analysis, generating more than one cost estimate by paper. Therefore, there is the possibility of ending with more cost estimates than papers reviewed. These cost estimates will be the base for the quantitative analysis of this paper. Details of this to follow.

A data extraction form included questions on: context (e.g. geographical study location), sampling and characteristics of sample (e.g. HIV/AIDS disease level), methods and results (e.g. type of costs analysis, year of prices, costs sources) and conclusions of the studies (e.g. results summary). Each abstract and paper selected was reviewed.

Three types of analyses were undertaken. First, summary descriptive statistics were used to describe the background (e.g. country, level of HIV/AIDS disease) and type of costs. Further quantitative analysis was based on the cost estimates reported by the papers including healthcare and non-healthcare costs. Average costs were calculated for each country according to the estimated resulting from the systematic literature review. Secondly, mean, standard deviations, 95% confident interval values calculated using STATA v9 were used to summarise mean costs and thirdly, comparison across countries was carried out. The average costs results provided by the papers and therefore results from this analysis rely on methods of calculation from each paper.

To aid comparative quantitative analysis, estimated costs were converted to €2010 using country-specific gross domestic product deflators [36] and World Bank purchasing power parities (PPP) [37]. Estimated costs values were multiplied by the coefficient of gross domestic product (GDP) for 2010 divided by the GDP of the particular year of the study, and then divided by the PPP conversion factor for 2010. If required, the exchange rates applied were from the European Central Bank, 2010. For those papers not reporting the year when the costs were calculated for, the publication was used. Those papers reporting the price along a two year interval (e.g. 1995–1996) the most recent year was used to update prices. In addition, to calculate the percentage that would involve treatment of HIV/AIDS out of the total healthcare expenditure, prevalence data - number of people who live with HIV/AIDS- and total healthcare cots on HIV/AIDS for each country were used. This percentage was calculated using 2008 as base year for all data.

## Results

**Figure 1:**
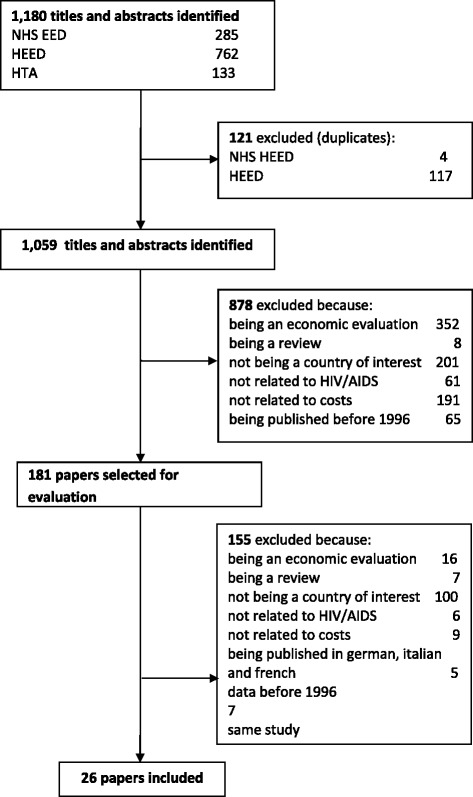
**Flowchart of study identification and selection.**

Figure 1 shows the flowchart for the identification of studies with reasons for exclusion. Overall, the search strategy identified 1,180 titles and abstracts from studies with potential for inclusion in this review. Based on the abstracts, 181 papers were ordered and manually reviewed. Of these 181 articles, 26 were included in this study. See Appendix 1 for details regarding included studies

### Description of cost studies on HIV/AIDS treatment

Tables 1 and 2 contain a description of the 26 articles included in the present study as well as the 76 cost estimates contained in these articles, more than one paper contained more than one cost estimate. The number of studies published on HIV/AIDS treatment over time is as follows: 8 articles (30.8%) were published before 2000, 11 articles first appeared between 2000 and 2004, and the remaining 7 (26.9%) were published between 2005 and 2009. The years with the greatest research output were 1998 and 2000 (6 and 4 articles published in each of these respective years).

**Table 1:** **Main characteristics of included studies**

**Country**	**Author**	**Publication year**	**Journal**	**Sample size**	**Focus (according papers’ authors)**	**Focus (according reviewers)**	**Costs approximation (according papers’ authors)**	**Costs approximation (according reviewers)**
Spain	Domingo JM et al.	1998	Oficial Journal Of The International AIDS Society	249	*No information*	Prevalence	*No information*	Bottom-up
Spain	Velasco M et al.	2000	HIV Medicine	155	*No information*	Prevalence	*No information*	Bottom-up
Spain	Mompó C et al.	2000	Gaceta Sanitaria	109	*No information*	Prevalence	*No information*	Bottom-up
Spain	Llibre-Codina JM et al.	2007	Enfermedades Infecciosas y Microbiologia Clínica	1.286	Prevalence	Prevalence	*No information*	Bottom-up
Spain	Velasco M et al.	2007	European Journal Of Internal Medicine	101	*No information*	Prevalence	*No information*	Bottom-up
Spain	Santolaya Perrín et al.	2008	The Annals Of Pharmacotherapy	144	*No information*	Prevalence	*No information*	Bottom-up
Spain	Lopez-Bastida et al.	2009	BIOMEDCENTRAL Health Services Research	572	Prevalence	Prevalence	Bottom-up	Bottom-up
Germany	Stoll M et al.	2002	Journal of Medical Research	168 (average 3 years)	*No information*	Prevalence	*No information*	Bottom-up
France	Mouton Y et al.	1997	Oficial Journal Of The International AIDS Society	7.749	*No information*	Prevalence	*No information*	Bottom-up
France	Rahmouni S et al.	1998	International Society For Pharmacoeconomics And Outcomes Research	1.558	*No information*	Prevalence	*No information*	Bottom-up
France	Peyron F et al.	1999	The Journal Of Pharmacy Technology	257	*No information*	Prevalence	*No information*	Bottom-up
France	Le Pen C et al.	2001	HIV Clinical Trials	500	*No information*	Prevalence	*No information*	Bottom-up
France	Yazdapnpanah Y et al.	2002	Antirretroviral Therapy	1.232	*No information*	Prevalence	Micro-costing	Bottom-up
Italy	Floridia M et al.	2000	HIV Clinical Trials	166	Incidence	Incidence	*No information*	Bottom-up
Italy	Garattini L et al.	2001	AIDS Care	483	*No information*	Prevalence	Micro-costing	Bottom-up
Italy	Sabbatini S, Cesari R.	2002	Clinical Drug Investigation	650	*No information*	Prevalence	*No information*	Bottom-up
Italy	Torti C et al.	2003	Health Policy	1.268 (average 4 years)	*No information*	Prevalence	*No information*	Bottom-up
Italy	Tramarin A et al.	2004	Pharmacoeconomics	74	*No information*	Prevalence	*No information*	Bottom-up
Italy	Merito M et al.	2005	Health Policy	5.422	*No information*	Prevalence	*No information*	Bottom-up
Italy	Hubben GAA et al.	2008	AIDS Care	119	Prevalence	Prevalence	Bottom-up	Bottom-up
United Kingdom	Beck EJ et al.	1998	Pharmacoeconomics	5.783	*No information*	Prevalence	Specific hospital activity related to specific hospital unit costs	Bottom-up
United Kingdom	NPMS Steering Group.	1998	International Journal Of STD & AIDS	6.002	Prevalence	Prevalence	*No information*	Bottom-up
United Kingdom	Beck EJ, Tolley K.	1998	International Journal Of STD & AIDS	5.708	Prevalence	Prevalence	*No information*	Bottom-up
United Kingdom	Sculpher MJ et al.	1998	Oficial Journal Of The International AIDS Society	*No information*	*No information*	Incidence	*No information*	Bottom-up
United Kingdom	Mullins CD et al.	2000	Clinical Therapeutics	4.817	*No information*	Prevalence	*No information*	Bottom-up
United Kingdom	Evans HER et al.	2009	Sexually Transmitted Infections	2.189	*No information*	Prevalence	*No information*	Bottom-up

**Table 2:** **Direct and non-direct costs variability intra country by patient (€ 2010)**

	Spain (n* = 9**)					Germany (n* = 3)					France (n* = 4**)					Italy (n* = 17**)					United Kingdom (n* = 15**)				
	**n**	**MEAN**	**SD**	**MIN**	**MAX**	**n**	**MEAN**	**SD**	**MIN**	**MAX**	**n**	**MEAN**	**SD**	**MIN**	**MAX**	**n**	**MEAN**	**SD**	**MIN**	**MAX**	**n**	**MEAN**	**SD**	**MIN**	**MAX**
**Healthcare costs**																									
Antiretrovirals	9	10.248,37	4.489,01	5.883,34	20.276,09	3	18.182,24	1.132,91	17.424,92	19.317,96	4	13.152,19	3.177,05	671,51	13.974,88	17	6.579,60	3.252,27	1.185,66	9.974,46	15	16.820,15	7.475,57	7.143,75	29.065,71
Other drugs	3	202,40	146,39	86,10	367,11	3	5.931,71	2.222,07	4.283,57	8.153,47	1	961,37	-	-	-	5	5.304,43	1.953,96	988,24	7.054,24	0	-	-	-	-
Hospitalization	6	985,68	997,51	31,09	2.621,26	3	6.258,58	3.058,57	3.980,94	9.316,08	2	1.629,00	298,80	1.575,33	2.460,77	11	1.178,43	884,20	67,17	3.966,24	9	7.241,7	6.249,54	1.884,59	21.275,59
Primary care	6	378,85	195,62	261,23	871,22	3	488,02	249,56	231,87	719,55	4	2.468,54	820,62	182,84	5.860,8	9	155,18	254,24	31,22	1.571,93	0	-	-	-	-
Acute care	6	97,29	52,19	52,61	211,45	0	-	-	-	-	0	-	-	-	-	0	-	-	-	-	0	-	-	-	-
Diagnostic tests	3	885,13	135,76	771,29	1.021,21	3	1.263,22	171,72	1.057,01	1.396,65	0	-	-	-	-	4	1.375,87	74,90	1.341,49	1.530,34	0	-	-	-	-
Other direct costs	2	33,24	50,66	4,78	78,33	3	104,41	26,23	84,91	130,63	1	4.099,84	-	-	-	10	713,93	1.160,36	230,55	10.111,36	9	7.060,13	4.384,84	3.245,49	13.052,02
Total heatlhcare costs ***	9	11.638,38	3.756,36	8.547,53	20.276,09	3	32.109,62	6.960,48	26.650,64	39.041,96	4	14.821,02	1.896,73	5.380,25	15.210,43	17	6.399,23	2.503,00	2.173,90	25.944,39	15	25.339,7	14.548,79	10.571,45	53.241,22
**Non-healthcare costs**																									
Occupational losses	3	5.661,18	1.871,58	4.045,37	7.152,04	0	-	-	-	-	0	-	-	-	-	3	1.353,56	609,90	788,55	1.986,69	6	5.613,33	4.564,90	1.722,51	11.962,60
Formal care	0	-	-	-	-	0	-	-	-	-	0	-	-	-	-	0	-	-	-	-	6	1.909,99	628,43	1.259,27	2.683,22
Informal care	0	-	-	-	-	0	-	-	-	-	0	-	-	-	-	0	-	-	-	-	6	1.816,30	531,32	1.451,24	2.588,28
Other non-direct costs	0	-	-	-	-	0	-	-	-	-	0	-	-	-	-	0	-	-	-	-	6	1.007,62	2.445,77	0	23.751,13
**Total non-healthcare costs**	3	5.661,18	1.871,58	4.045,37	7.152,04	0	-	-	-	-	0	-	-	-	-	3	1.353,56	609,90	788,55	1.986,69	6	10.347,24	7.448,33	4.443,02	23.751,13

* = number of cost estimates included in the analysis;** = In France, Spain, Italy and United Kingdom, we found 22 cost estimates, originated by 8 different studies, that: (a) did not have similar methodology than the rest of studies, so, there were indicen studies (when most part of the studies were prevalence studies); and, (b) did not incorporate antiretrovirals costs and another type of cost (so, these studies either incorporate antirretroviral costs or any other type of cost). For this reason, authors decided to include in this comparative intra country analysis with only 61 estimates and do not incorporate studies such as Domingo et al. (1998), Evans et al. (2009), Floridia et al. (2000), Llibre-Codina et al. (2007), Mompó et al. (2000), Rahmouni et al. (1998), Sculpher et al. (1998) and, Yazdanpanah et al. (2002); and, *** Total healthcare costs are not equal to the summ of means of each different type of costs due to: (i) not all the different types of specified costs in this list were included in the different studies; and, y (ii) the average cost has been weighted by the sample size for each study.

Of all the articles reviewed, 26.9% investigated HIV/AIDS treatment costs in Spain (7 articles), while other countries include Italy (7 articles), the United Kingdom (6 articles), and France (5 articles). Only 1 of the articles was published by Germany. Although all of the cost studies analyzed HIV/AIDS treatment in European countries, 4 (15.38%) were written by authors based in the United States, a country with a long-standing tradition and experience in HIV/AIDS research.

The sample studied herein comprises 25 of the 26 articles accessed (96.15% of the total). Of all the cost estimates in the articles (76 treatment costs), 21 were based on studies that included fewer than 150 patients. The average number of patients included in all of the studies was 1,743; the standard deviation (SD) was near the average (2,120); the median value was 1,127 patients. The discrepancy between the mean and median values is due to the sample-size dispersion in the articles reviewed; indeed, we found patient samples that ranged from 37 (lowest) to 6,002 (highest).

Thirty-two of the 76 cost estimates report patient ages (average age: 31.78 years), and 40 provide information on distribution by sex (78% males). In 28 of the 76 cost estimates (36.84%), the primary means of transmission was intravenous-drug use, while in 36.84% of the cases, the disease was transmitted sexually. Transmissions via blood transfusion (n = 8; 10.53%) and in patients with hemophilia (n = 7; 9.21%) accounted for the lowest number of HIV/AIDS infections. Fifteen of the cost estimates (19.73%) reported average viral load values, expressed in copies per milliliter (average viral load: 16,836.27 copies/mL, SD 17,213.01). The lowest number of copies per milliliter was 3, and the highest was 49,069. Thirty-eight (50%) of the 76 cost estimates provided mean CD4+/mm3 lymphocyte counts; the mean count for all observations was 290.16 CD4+/mm3, with an SD of 159.76 CD4+/mm3. The lowest lymphocyte count in all the studies was 14 CD4+/mm3, and the highest was 713 CD4+/mm3. The study that reports a lymphocyte count of 14 CD4+/mm3 clarifies that the patients included were in an advanced stage of the disease and had lymphocyte counts below 50 CD4+/mm3. Only 3 of the studies provide data on median CD4 counts, with these data appearing in 9 different estimates. All the other reports express CD4 data as mean values.

Twenty-three of the 76 cost estimates (30.26%) do not indicate the developmental stage of the illness in the patients included. In 11.84% of the estimates, all of the patients studied were asymptomatic, while 11.84% of the calculations were based on patients who had progressed to AIDS. In 11 of the estimates the patients had AIDS, and in 3 the individuals were AIDS patients who reported whether or not they had had a previous AIDS-related event. Twenty-one estimates (27.63%) used alternative means of describing the developmental stage of the disease, such as the CDC classification system and disease severity, or according to whether or not they had progressed to AIDS and whether or not they had participated in a study that included antiretroviral therapy, or depending on diagnostic status or per type of disease accompanying AIDS, etc.

**Figure 2:**
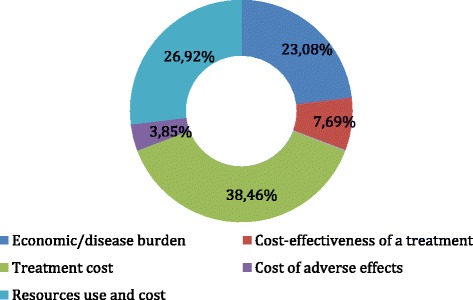
**Distribution of study types.**

In terms of type of cost study (see Figure 2), of the 26 articles analyzed, 1 examined the cost of the adverse effects of the treatment, and 2 of the studies analyzed the treatment costs and independently assessed the effectiveness of the treatment without comparing the two. However, most of the studies analyzed the cost of treatment or the use and the cost of the resources for treating HIV/AIDS.

Ten of the 26 articles (38.46%) specifically mentioned the point of view from which the study was conducted. Of these 10 articles, 7 (26.92%) examined the issue from the perspective of funding sources, 1 analysis (3.85%) focused on the point of view of the provider, and 2 articles (7.69%) adopted a social perspective. In 84.61% of the articles that did not make specific reference to the analytical perspective employed, this information was gleaned from the details of the text. It was not possible to ascertain the analytical perspective in 4 of the articles. Fifty percent (n = 13) of the articles approached the issue from the perspective of the funding source.

Only 6 articles expressly indicate the approach used for the cost study. One article (3.85%) focuses on incidence, while 5 base their analysis on prevalence (19.23%). While 76.92% of the authors of the remaining articles (n = 20) did not specify the approach used, a review of the works revealed that 92.31% of the articles (n = 24) are based on prevalence, while 7.69% approach the topic from the viewpoint of incidence.

All the articles used a bottom-up approach (cost calculation progressing from most detailed to most general) for their cost analyses, although only 19.23% of the articles (n = 5) make explicit reference to this information. Eleven of the articles (42.31%) used the euro as the currency of reference for cost analyses, followed by 7 articles (26.92%) which expressed costs in pounds sterling. Three articles used the French franc, and 1 study used the Spanish peseta. (These works predate the introduction of the euro.) Although all the articles reviewed in the present study deal with European countries, 4 of them (15.38%) calculated costs in US dollars.

Costs are expressed according to pre-2000 price levels in 57.69% of the articles (n = 15), while only 5 used post-2000 prices for their calculations (23.08%). In 23.08% of the articles (n = 6), the year used for price calculations was not provided.Costs were expressed in daily, monthly, mean, and annual prices per patient, although the necessary transformations and calculations were carried out to allow for uniform, per-patient cost components. Sixty-two of the cost estimates (81.58%) only calculated medical costs, while 6 (7.9%) only included non-medical costs. Eight of the estimates included both cost types.

Only 65 of the 76 estimates of the cost of treating HIV/AIDS patients (86%) specified the temporal horizon they used. Thirteen of the studies (20%) were conducted over more than 1 year, while 40 analyses used a 1-year temporal horizon (61.53%). All other analyses took place over less than 1 year. The mean (median) temporal horizon used in the studies was 75.05 (52) weeks.

Regarding sources of funding, 15 of the 26 works provided this information. Private-sector funding was used in 26.92% of the cases (n = 7), and public-sector funding was used in 23.08% (n = 6). Merck and GSK funded more studies than any other firms that market therapy, backing 3 and 2 studies, respectively.

### Health care costs

Of the 70 estimates that calculated healthcare costs, 74.29% used primary data sources (clinical trials) (n = 52) to obtain information on resource use and costs. Only 12 estimates (17.14%) used secondary data sources (data gathered from bibliographical references or published studies). The remaining 6 estimates (8.57%) used a combination of primary and secondary sources for their data.

Concerning medical costs, 67 of the 70 estimates made specific reference to the type of direct costs studied. Fifty-nine estimates referred to the costs of antiviral medications (77.63%), 48 reports included hospitalization costs (63.16%), and 42 (55.26%) detailed the costs of primary and outpatient care. The cost of diagnostic tests is analyzed in 27.63% of the estimates (n = 18), and 7.89% (n = 6) analyze the cost of emergency care. The total health care costs are those specified and calculated in the study. Some of these cost calculations are the sum of the columns containing the different cost items, although at times this is not the case.

### Non-health care costs (lost working time and costs of formal and informal care)

Of the 14 estimates of non-healthcare costs, 8 (57.14%) used primary sources to gather data on resource use and costs, and 6 other estimates (42.86%) used an approach that combined primary and secondary sources. In 85.71% of the estimates of non-health care costs (n = 12), the calculations include lost work time in HIV/AIDS patients, while the remaining 2 estimates compute the costs of formal (paid) and informal (unpaid) care required by these patients. The total non-health care costs are those specified and calculated in the study. Some of these cost calculations are arrived at by adding together the columns containing the different cost items, although at times this is not the case.

### Primary country-specific costs: intra- and inter-country variability

The mean estimated annual cost of HIV/AIDS treatment in the European countries described as part of this study appears in Table 3. To allow for proper comparison, the results appearing in this table were obtained from studies adopting a similar methodology. All of the figures have been updated by taking 2010 as a reference year. The primary findings are as follows:

**Table 3:** **Direct and non-direct costs variability intra country per patient according to phase of the disease and lymphocytes level (€ 2010)**

***Total healthcare costs***	Spain (n* = 9)					Germany (n* = 3)					France(n* = 4)					Italy (n* = 17)					United Kingdom (n* = 15)				
	**n**	**MEAN**	**SD**	**MIN**	**MAX**	**n**	**MEAN**	**SD**	**MIN**	**MAX**	**n**	**MEAN**	**SD**	**MIN**	**MAX**	**n**	**MEAN**	**SD**	**MIN**	**MAX**	**n**	**MEAN**	**SD**	**MIN**	**MAX**
According to phase of the disease (**)																									
Asymptomatic phase	1	8.547,53	-	-	-	0	-	-	-	-	0	-	-	-	-	1	2.173,9	-	-	-	5	1.4991,73	3.493,29	10.571,45	18.290,76
Symptomatic phase	1	10.173,81	-	-	-	0	-	-	-	-	0	-	-	-	-	0	-	-	-	-	5	19.222,25	4.437,73	12.131,42	22.945,24
AIDS phase	2	11.032,38	1.058,39	10.272,01	11.768,99	0	-	-	-	-	0	-	-	-	-	1	15.182,96	-	-	-	5	42.968,55	12.487,71	23.155,18	53.241,22
**According to lymphocytes level (CD4 cells)**																									
CD4s < 200	0	-	-	-	-	0	-	-	-	-	0	-	-	-	-	3	19.252,63	5.840,26	15.182,96	25.944,39	3	49.326,37	6.250,58	42.117,73	53.241,22
200 < CD4s < 500	3	9.922,42	750,11	8.849,42	10.272,01	3	32.109,62	6.960,48	26.650,64	39.041,96	1	13.396,55	-	-	-	2	12.227,88	2.062,68	9.670,24	13.059,63	9	21.445,86	9.572,81	11.889,08	45.811,29
**CD4s > 500**	0	-	-	-	-	0	-	-	-	-	0	-	-	-	-	3	6.399,34	2.369,71	2.173,9	10.363,14	0	-	-	-	-

* = number of cost estimates included in the analysis; and, ** = there are some studies that do not specify the phase of the disease from patients.

In Spain, the estimated treatment cost per patient-year is €11,638, with an SD of €3,756. The approximate annual cost of treating these patients in Germany is €32,110 (SD €6,960). For France, the estimated per-person annual treatment cost is €14,821 (SD €1,897). HIV/AIDS treatment in Italy costs an average of about €6,399 per patient (SD €2,503). Lastly, it was found that the mean cost of this treatment per patient-year in the United Kingdom was €25,340 (SD €14,549).

The results appearing in Table 3 demonstrate a high degree of inter-country variability, with Spain, France, and Italy showing substantially lower annual per-patient costs than those found for Germany and the United Kingdom.

Studies carried out in different countries using similar methodologies produced similar findings in this regard, indicating that drugs—mainly antiretroviral medications—are the primary medical-care cost component, followed by hospitalization costs, costs for outpatient and primary-care consultations, and the costs of diagnostic tests.

In terms of health care costs, the lowest cost of antiretroviral treatment was found in Italy, followed by Spain and France. It was observed that the cost of medications in Germany and the United Kingdom are well above those of the 3 aforementioned countries. It must also be pointed out that hospitalization costs are much lower in Spain than in the rest of the countries studied, and a significant difference was observed between inpatient-care costs in Spain, Italy, and France compared to those in Germany and the United Kingdom.

In light of the great disparity of medical-care costs between countries, the country-specific costs incurred were calculated, and these can be seen in Table 4. These data indicate which stage of the disease is being treated and the lymphocyte counts of the patients receiving the treatment. The only country in which the studies reviewed included disease-phase information is the United Kingdom; the absence of this information for all the other countries is noteworthy. We can only state that in Spain and the United Kingdom, total medical-care costs rise progressively in step with increasing disease stages. Results according to the lymphocyte counts of patients are also presented. The United Kingdom and Italy are the 2 countries that provide the most detailed information on this indicator. The lack of such information from the rest of the countries is once again noteworthy. In the United Kingdom and Italy, a progressive cost increase is seen in patients with lower lymphocyte counts, that is, patients who are more susceptible to have other illnesses. There is also a great disparity in total medical costs for patients with lymphocyte counts between 200 CD4+/ mm3 and 500 CD4+/ mm3, although the reason for this variation is unclear.

**Table 4:** **People who live with HIV/AIDS**

**Year 2007**	**Base**	**Lower quote**	**Upper quote**
**Spain**	140.000	80.000	230.000
**Germany**	53.000	31.000	97.000
**France**	140.000	78.000	240.000
**Italy**	150.000	110.000	210.000
**United Kingdom**	77.000	37.000	160.000

Source: ONUSIDA, 2008.

We carried out an analysis to establish whether a link exists between higher total care costs and greater number of years elapsed since the calculation of these costs, although no such link was found. Inter-country differences in total care costs may be caused by price differences, differing frequency of hospital use, or the cost of medications; however, more information would be required from the authors of the different studies in order to reach a conclusion.

The results of the works analyzed together with information on medical-care spending and official figures of HIV/AIDS prevalence in the 5 countries studied were extrapolated in order to estimate the total annual cost of treating people with HIV/AIDS. Since the data available on prevalence are from 2007 [20], and since data on medical-care costs as a percentage of gross domestic product are available only up to 2008, an estimate was made using 2008 as the base year, as the prevalence figures showed slight year-to-year variation. Table 5 contains the total estimated medical costs for each country as well as the percentage devoted to HIV/AIDS treatments relative to overall medical spending. This estimate was calculated by applying the conservative scenario by which it is assumed that 25% of HIV-positive individuals are unaware that they are infected and, therefore, are not receiving treatment.

**Table 5:** **Total healthcare cost of treatments of HIV/AIDS and% of the total health cost from each country (€2008)**

	**Total healthcare costs (million€)**			**Healthcare cost as a % of the total health cost**		
	**Base**	**Lower quota**	**Higher quote**	**Base**	**Lower quota**	**Higher quota**
**Spain**	1.222,16	698,38	2.007,84	1,25%	0,72%	2,06%
**Alemania**	1.253,89	733,40	2.294,85	0,48%	0,28%	0,88%
**France**	1.124,74	626,64	1.928,12	0,52%	0,29%	0,88%
**Italy**	695,07	509,72	973,10	0,49%	0,36%	0,68%
**United Kingdom**	1.406,57	675,89	2.922,75	0,90%	0,43%	1,86%

Source: Own elaboration from the data provided by different sources.

According to our calculated average healthcare cost of treatments of HIV/AIDS resulting from the quantitative analysis, the cost of HIV/AIDS in Spain represents 1.25% of overall medical-care spending, while total HIV/AIDS-treatment spending amounts to 0.49% in Italy, 0.52% in France, and 0.9% in the United Kingdom. In Germany, despite the country’s low HIV/AIDS prevalence, 0.48% of total medical-care spending is devoted to treating the disease. When compared to other health problems, these figures are high given the low prevalence of HIV/AIDS in terms of population. If we consider that almost all of the medical-care spending for HIV/AIDS treatment in Spain is publicly funded, the percentage of HIV/AIDS medical costs rises to 1.73% of the total public-sector spending.

## Discussion and conclusions

Despite the evidence demonstrating that the social and economic cost of HIV/AIDS is quite substantial, there are still areas where knowledge of this issue is scant. This review contributes in analysing the effort done in the last decade to measure the cost of illness of this disease and highlights the way forward for advancing in this area. There is a great paucity of information available on the non-medical costs incurred due to HIV/AIDS treatment. The non-medical cost components that were identified and assigned a monetary value comprised lost working time, formal and informal care, and disability payments. Although no studies of this nature have been carried out in France or Germany, research in Spain, Italy, and the United Kingdom has revealed that while these values are lower than medical-care costs, they are substantial sums nonetheless.

While there appears to be little doubt that prolonging the life expectancy of carriers of the virus who live in high-income countries increases the cost of treating each patient throughout his or her life, there is still no international consensus on whether or not the advances made in treating the disease have increased the annual cost of caring for these individuals. These costs may vary significantly from one country to another, and there may be great inter-country disparity in the economic impact of HIV/AIDS treatment depending on the unit costs of the treatment as well as the epidemiology of the disease in a given country. Aside from the health care costs of HIV/AIDS treatment, a number of social costs exists which may have a substantial impact on studies of the disease, such as lost work time in HIV-infected individuals.

The studies reviewed for the present work point to a substantial variation in the cost of treating patients with HIV/AIDS since the advent of highly active antiretroviral therapy (HAART). Our endeavor to properly compare the estimates published—including the different cost items factored into care costs—was a complex task due to the inconsistencies in the components included as well as the lack of explicit information in the articles. These difficulties appear to be common in the literature on HIV/AIDS. Indeed, Levy et al. [26] came to a similar conclusion in their review article on the medical-care costs of HIV/AIDS.

In the comprehensive review by Beck et al. [3], the authors highlight the need to increase the body of evidence that can be used to more accurately estimate the overall economic impact of HIV/AIDS infection. The authors found only 3 studies published after 1996. The present article, however, which focuses on the European context, analyzes 26 articles. This number is indicative of the great effort and the high interest in estimating the global cost of this illness.

The methodological approach used in the articles reviewed is predominantly bottom-up (i.e., based on patient samples), as there are scant sources of reference (e.g., registries, databases) of a national or international scope which would allow for a top-down approach to be used.

In addition, most studies approach the issue by focusing on prevalence; incidence-based studies are more scarce. This result is common in cost of illness studies in other areas of treatment, as incidence-based approaches have more stringent requirements regarding the information needed to carry out the analysis; indeed, incidence-based studies require life-expectancy and cost estimates incurred throughout the natural course of the disease.

It must be pointed out that the figures appearing in Table 3 do not include all of the studies reviewed, but rather those that employ a comparable methodological approach. The reason for this is, on the one hand, the different objectives and study designs of the works reviewed, and on the other, the fact that the methodological approaches used in HIV/AIDS cost-of-illness studies have improved. Regarding the first reason, it would be unreasonable to compare incidence-based and prevalence-based studies, just as it would be unreasonable to compare studies that focus on the cost of treating adverse effects (very concrete objective) to HIV/AIDS cohort studies that seek to determine the costs incurred throughout the study period (more general objective). As for the second reason, there is ample room for improvement in the studies revised, as many of them fail to adequately detail such essential elements as an explicit description of the vantage point used (e.g., social, funding, provider), the approach (e.g., incidence, prevalence), and other methodological aspects. In addition, the cost components are not uniformly disaggregated, and one can find some studies in which pharmaceutical treatment is included under hospital care, while others compute drug therapy under outpatient resources. Such methodological heterogeneity increases the difficulty of conducting inter-study comparisons.

While the number of studies on the medical costs (direct costs) of HIV/AIDS treatment has increased considerably, the present work has found that few publications take non-medical costs into account. In their 2004 study, Liu et al. [37] emphasized the need for studies that include non-medical aspects, although in the years since their work the output of this type of research has continued to be low.

Although few studies have set out to estimate the non-medical costs of HIV/AIDS in high-income countries (mainly lost work time but also the cost of formal and informal care), it is worth noting that other works have highlighted the substantial impact that HIV/AIDS had on work engagement among carriers of the disease in the years before effective treatments were made available [27–30]. Also, later studies have clearly indicated that the advances in therapy that have been achieved since the mid-1990s have had a major impact on this issue, and a number of studies have published evidence of this effect [6,31–35]. These studies have not been included in the present review since they provide information on changes in the probability of being employed of HIV + people without assigning a monetary value to this change. However, we should stress that the aforementioned studies report improvements in the labor situation of people with HIV/AIDS, this does not mean that the general population has also experienced such an improvement. Significant differences exist between the 2 groups, and these differences are more pronounced at greater levels of health deterioration, and differences also exist in terms of the patterns or habits of the individuals studied as revealed by the means through which they were infected [6,38].

In summary, this review shows the profound impact that HIV/AIDS-treatment costs have in the countries studied. Our study also shows that the methodology used in many of the studies carried out in this field leaves ample room for improvement. Lastly, although there has been a marked increase in the number of investigations on medical-care costs in recent years, there is still a great amount of work to be done toward applying the social perspective to these cost analyses [39]. In addition to calculating the medical and non-medical costs associated with HIV/AIDS, studies of this nature should reflect the social burden that lies outside the healthcare realm but which nonetheless is borne by persons who are not carriers of the disease. The impact of this disease may fall on society as a whole.

## Appendix 1. Papers included in the systematic literature review (26 papers)

Beck EJ, Tolley K: **Financing HIV service provision in England: estimated impact of the cost of antirretroviral combination therapy.** International Journal of STD & AIDS 1998, **9**(9):512–517.

Beck EJ, Tolley K, Power A, Mandalia S, Rutter P, Izumi J, Beecham J et al.: **The use and cost of HIV service provision in England in 1996.** Pharmacoeconomics 1998, **14**(6): 639–652.

Domingo JM, Guardiola J, Ris J, Nolla J: **The impact of new antirretroviral regimes on HIV-associated hospital admissions and deaths.** AIDS 1998, **12**(5):529–543.

Evans HER, Tsourapas A, Mercer CH, Rait G, Bryan S, Hamill M, et al.: **Primary care consultations and costs among HIV-positive individuals in UK primary care 1995–2005: a cohort study.** Sexually Transmitted Infections 2009, **85**(7):543–549.

Floridia M, Massella M, Bucciardini R, Perucci CA, Rossi L, Tomino C et al.: **Hospitalizations and costs of treatment for protease inhibitor-based regimens in patients with very advanced HIV-infection (CD4 < 50/mm3).** HIV Clinical Trials 2000, **1**(2):9–16.

GarattiniL, Tediosi F, Di Cintio E, Yin D, Parazzini F: **Resource utilization and hospital cost of HIV/AIDS care in Italy in the era of highly active antirretroviral therapy.** AIDS Care 2001, **13**(6):733–741.

Hubben GAA, Bishai D, Pechlivanoglou P, Cattelan AM, Grisetti R, Facchin C, et al.: **The societal burden of HIV/AIDS in Northern Italy: An analysis of costs and quality of life.** AIDS Care 2008, **20**(4) :449–455.

Le Pen C, Rozenbaum W, Downs A, Maurel F, Lilliu H, Brun C: **Effect of TARGA on health status and hospital costs of severe HIV-infected patients: a modeling approach.** HIV Clinical Trials 2001, **2**(2):136–145.

Llibre-Codina JM, Casado-Gomez MA, Sánchez-de la Rosa R, Pérez-Elías MJ, Santos-González J, Miralles-Álvarez C et al.: **Costes de la toxicidad asociada a los análogos de nucleósidos inhibidores de la transcriptasa inversa en pacientes con infección por el VIH-1.** Enfermedades Infecciosas y Microbiologia Clínica 2007, **25**(2): 98–107.

Lopez-Bastida J, Oliva-Moreno J, Perestelo-Perez Ll, Serrano-Aguilar P: **The economic costs and health-related quality of life of people with HIV/AIDS in he Canary Islands, Spain.** BMC Health Services Research 2009, **9**:55–63.

Merito M, Bonaccorsi A, Pammolli F, Riccaboni M, Baio G, Arici C, et al.: **Economic evaluation of HIV treatments: The I.C.O.N.A. cohort study.** Health Policy 2005, **74**(3):304–313.

Mompó C, Abbas I, Santín M, Rovira J, Antón F, Tomás C, et al.: **La utilización de recursos sanitarios en pacientes infectados por el virus de la immunodeficiencia humana: creación de una base de datos y obtención de resultados de costes.** Gaceta Sanitaria 2000, **14**(1):39–47.

Mouton Y, Alfandari S, Valette M, Cartier F, Dellamonica P, Humbert G et al.: **Impact of protease inhibitors on AIDS-defining events and hospitalizations in 10 French AIDS reference centres.** AIDS 1997, **11**(12):101–105.

Mullins CD, Whitelaw G, Cooke JL, Beck EJ: **Indirect cost of HIV infection in England.** Clinical Therapeutics 2000, **22**(11):1333–1345.

NPMS Steering Group: **Changing cost of English HIV service provision 1996–1997.** International Journal of STD & AIDS 1999, **10**(6):357–362.

Peyron F, Rahmouni S, Flori A, Moreau J, Charbit J-J, Buès-Charbit M, et al.: **Impact of protease inhibitors on drug use and cost for hospital patients with HIV.** Journal of Pharmacy Technology 1999, **15**(6):212–218.

Rahmouni S, Peyron F, Flori A, Moreau J, Charbit JJ, BuesCharbit M, Balansard G: **Pharmacoeconomic efficiency of the use of protease inhibitors for hospital inpatients with AIDS. ISPOR, Inaugural European Conference (Cancer and Aids). **1998. Cologne, Germany.

Sabbatani S, Cesari R: **Cost assessment of antirretroviral drugs used in the treatment of patients with HIV infection. Focus on non-nucleoside reverse transcriptase inhibitors.** Clinical Drug Investigation 2002, **22**(4):253–262.

Santolaya Perrín R, García López FJ: **Incremental drug treatment cost in HIV-positive patients in industry-sponsored clinical trials.** The Annals of Pharmacotherapy 2008, **42**(11):1586–1591.

Sculpher MJ, Gibb D, Ades AE, Ratcliffe J, Duong T: **Modelling the costs of paediatric HIV infection and AIDS: comparison of infected children born to screened and unscreened mothers.** AIDS 1998, **12**(11):1371–1380.

Stoll M, Claes C, Schulte E, Graf von der Schulenburg JM, Schmidt RE: **Direct costs for the treatment of HIV-infection in a German cohort after the introduction of TARGA.** European Journal of Medical Research 2002, **7**(11):463–471.

Torti C, Casari S, Palvarini L, Quiros-Roldan E, Moretti F, Leone L, et al.: **Modifications of health resource-use in Italy after the introduction of highly active antirretroviral therapy (TARGA) for human immunodeficiency virus (HIV) infection. Pharmacoeconomic implications in a population-based setting.** Health Policy 2003, **65**(3):261–267.

Tramarin A, Campostrini S, Postma MJ, Calleri G, Tolley K, Parise N et al.: **A multicentre study of patient survival, disability, quality of life and cost of care. Among patients with AIDS in Northern Italy.** Pharmacoeconomics 2004, **22**(1):45–53.

Velasco M, Gómez A, Fernández C, Pérez-Cecilia E, Téllez MJ, Roca V, et al.: **Economic impact of HIV protease inhibitor therapy in the global use of health-care resources.** HIV Medicine 2000, **1**(4):246–251.

Velasco M, Losa JE, Espinosa A, Sanz J, Gaspar G, Cervero M, et al.: **Economic evaluation of assistance to HIV patients in a Spanish hospital.** European Journal of Internal Medicine 2007, **18**(5):400–404.

Yazdanpanah Y, Goldie SJ, Losina E, Weinstein MC, Lebrun T, Paltiel AD, et al.: **Lifetime cost of HIV care in France during the era of highly active antirretroviral therapy.** Antirretroviral Therapy 2002, **7**(4):257–266.
